# Denoising and Baseline Correction Methods for Raman Spectroscopy Based on Convolutional Autoencoder: A Unified Solution

**DOI:** 10.3390/s24103161

**Published:** 2024-05-16

**Authors:** Ming Han, Yu Dang, Jianda Han

**Affiliations:** 1 Institute of Robotics and Automatic Information System, College of Artificial Intelligence, Nankai University, Tianjin 300350, China; 2120210395@mail.nankai.edu.cn (M.H.); hanjianda@nankai.edu.cn (J.H.); 2 Engineering Research Center of Trusted Behavior Intelligence, Ministry of Education, Nankai University, Tianjin 300350, China; 3 Tianjin Key Laboratory of Intelligent Robotics, Nankai University, Tianjin 300350, China; 4 Institute of Intelligence Technology and Robotic Systems, Shenzhen Research Institute of Nankai University, Shenzhen 518083, China

**Keywords:** autoencoder, convolutional neural network, preprocessing

## Abstract

Preprocessing plays a key role in Raman spectral analysis. However, classical preprocessing algorithms often have issues with reducing Raman peak intensities and changing the peak shape when processing spectra. This paper introduces a unified solution for preprocessing based on a convolutional autoencoder to enhance Raman spectroscopy data. One is a denoising algorithm that uses a convolutional denoising autoencoder (CDAE model), and the other is a baseline correction algorithm based on a convolutional autoencoder (CAE+ model). The CDAE model incorporates two additional convolutional layers in its bottleneck layer for enhanced noise reduction. The CAE+ model not only adds convolutional layers at the bottleneck but also includes a comparison function after the decoding for effective baseline correction. The proposed models were validated using both simulated spectra and experimental spectra measured with a Raman spectrometer system. Comparing their performance with that of traditional signal processing techniques, the results of the CDAE-CAE+ model show improvements in noise reduction and Raman peak preservation.

## 1. Introduction

Raman spectroscopy is a non-destructive measurement method that is used in various fields, such as analytical chemistry [1], biomedical application [2], and geology [3]. A Raman spectrum can indicate species of molecules owing to its characteristic peaks. The Raman spectrum is collected by a Raman spectrometer when a laser source is focused onto the sample and scattered from the sample. However, the Raman effect is an inherently weak effect—typically 10^−8^ of the intensity of the incident exciting radiation [4]. This makes Raman spectroscopy very sensitive to factors such as fluorescence background, Gaussian noise, and cosmic rays. In addition to the preparation of sensitive measurement devices, various signal processing procedures have been applied to extract useful information from the collected spectra.

The two most common preprocessing procedures in spectroscopy are noise reduction and baseline correction. For the denoising algorithm, there are several methods, such as wavelet threshold denoising (WTD) [5], Savitzky–Golay (SG) filtering [6], local weighted regression, and so forth. Baseline correction algorithms include piecewise linear fitting [7], adaptive iteratively reweighted penalized least squares (airPLS), iterative polynomial fitting, and so on. However, the effectiveness of these algorithms depends on the selection of parameter settings; processing results obtained with different parameters can vary, and the choice of parameters largely relies on the experience of the operator. Denoising algorithms can negatively impact spectral features [8,9]. This is especially true for sharp Raman peaks. On the other hand, baseline correction algorithms often reduce Raman peak intensity. This reduction becomes particularly problematic in cases of complex baselines and broad peaks.

In recent years, significant progress has been made in the field of spectral preprocessing using deep learning. Wang et al. applied backpropagation (BP) neural networks for spectral denoising and compared results to optimal wavelet threshold methods. The results demonstrate that the BP neural network not only simplifies the complex optimization of parameters but also achieves similar effectiveness to optimal wavelet transform [10]. Researchers have also utilized convolutional neural networks (CNNs) with customized loss functions to effectively smooth noise while striking a balance between smoothing of the spectrum and preserving peak values [11]. Additionally, Pan et al. have demonstrated that deep learning (DL) networks can outperform wavelet denoising methods while maintaining a high degree of similarity to the original spectral signal [12]. Liu et al. developed Baseline Recognition Networks (BRNs), using adversarial networks and deep residual learning for precise automatic baseline correction in spectroscopy, effectively eliminating the requirement for manual parameter adjustments [13]. Schmidt et al. deployed a convolutional neural network (CNN) for peak detection and baseline correction, achieving lower mean absolute error across various signal-to-noise ratios compared to the traditional wavelet transform method [14]. Furthermore, researchers have developed preprocessing networks using convolutional neural networks to integrate cosmic ray removal, the denoising method, and baseline correction into a single network [15]. Most current denoising research emphasizes improving the signal-to-noise ratio and adaptability, and baseline correction efforts concentrate on efficiency and reducing parameter dependence. However, the preservation of Raman peak intensities is overlooked in both denoising and correction.

Convolutional autoencoders (CAEs) have emerged as a powerful tool in image processing, combining the strengths of autoencoders and convolutional neural networks (CNNs). CAEs can effectively remove noise and preserve fine details, which are widely used for image preprocessing and feature extraction [16,17,18,19]. In this paper, we propose a unified solution for preprocessing of Raman spectroscopy data that includes two specialized models, namely a convolutional denoising autoencoder (CDAE) for denoising and a convolutional autoencoder (CAE+) for baseline correction. The novelty of these two methods lies in the integration of the convolutional layers and a comparison function into the convolutional autoencoder, which enhances the performance of denoising and baseline correction. Specifically, the CDAE model incorporates two additional convolutional layers in its bottleneck layer for enhanced noise reduction. The CAE+ model incorporates a comparison function after the decoder specifically designed for effective baseline correction. Herein, we demonstrate that our algorithms not only enhance preprocessing capabilities but also effectively preserve the intensity of Raman peaks in the spectrum.

## 2. A Unified Solution for Preprocessing Algorithms

### 2.1. Denoising Autoencoder

**Figure 1:**
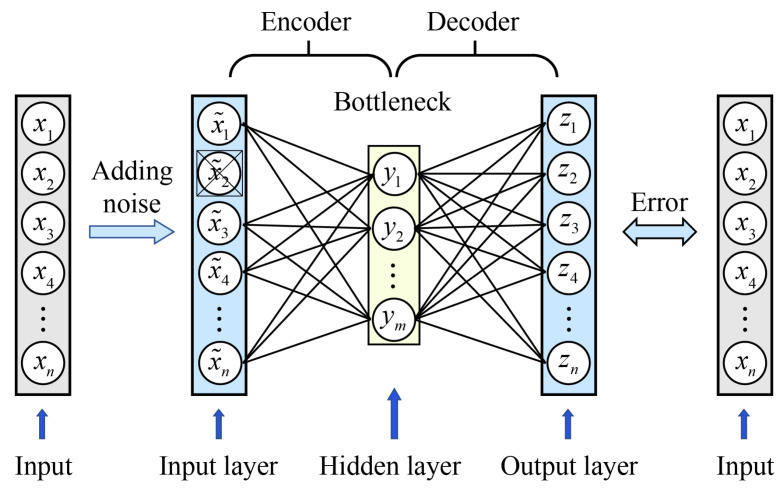
The structure of the autoencoder.

Autoencoders are a type of unsupervised learning algorithm that have been developed since the 1980s; they learn a compressed representation of data through an encoding and decoding process. In 2006, Hinton and Salakhutdinov significantly enhanced the capabilities of autoencoders in terms of dimensionality reduction and feature learning with their seminal work [20], surpassing the traditional effects of principal component analysis. The denoising autoencoder is an extension of the traditional autoencoder proposed by Vincent et al. [21]. The denoising autoencoder, during its training phase, uses synthetic data as input. This process enhances its robustness and the quality of feature representations by intentionally adding extra noise. Consequently, this strategy improves the model’s generalization ability, enabling it to effectively denoise and reconstruct the original data accurately. Figure 1 depicts the basic structure of a denoising autoencoder, which consists of the following three primary components: an input layer, a hidden layer, and an output layer. The hidden layer can be divided into the encoder and the decoder. The bottleneck connects an encoder and decoder in the central position and has the lowest dimensionality.

First, the input layer ($\tilde{x}$) is obtained by adding noise to the initial input (x). Dropout is applied to prevent overfitting. Then, the corrupted input ($\tilde{x}$) is mapped to a hidden representation via an encoder function. This hidden representation (y) is defined as follows:

(1) $$y=f_{\theta }\left(\tilde{x}\right)=h\left(W\tilde{x}+b\right)$$

where $\tilde{x}\in {\left[0,1\right]}^{n}$, and $y\in {\left[0,1\right]}^{m}$. W is a m×n weight matrix, and b is a bias vector. *h* is a nonlinear transfer function, and θ={W,b} is the parameter space of the $f_{\theta }$ function.

Then the derived hidden representation (y) is then mapped back to the output (z), as denoted by the decoder function, which is defined as follows:

(2) $$z=g_{\phi }\left(y\right)=h\left(W^{\prime }y+b^{\prime }\right)$$

where $z\in {\left[0,1\right]}^{n}$, and $\phi =\{W^{\prime },b^{\prime }\}$ is the parameter space of the $g_{\phi }$ function.

Thus, each corrupted input (${\tilde{x}}^{\left(i\right)}$) is mapped to a corresponding $y^{\left(i\right)}$, then reconstructed to ${\tilde{z}}^{\left(i\right)}$, where *i* indicates indexes of every training sample. In order to minimize the error between the output (z) and the initial input (x), the optimal parameters of this model are defined as follows:

(3) $$\begin{array}{cc} \theta^{*},\phi^{*} & =\arg\min_{\theta ,\phi }\frac{1}{n}\sum_{i=1}^{n}L\left(x^{\left(i\right)},{\tilde{z}}^{\left(i\right)}\right)\\ \; & =\arg\min_{\theta ,\phi }\frac{1}{n}\sum_{i=1}^{n}L\left(x^{\left(i\right)},g_{\phi }\left(f_{\theta }\left({\tilde{x}}^{\left(i\right)}\right)\right)\right) \end{array}$$

where *L* is a loss function.

Finally, the result of this model is derived by substituting the parameters ($\theta^{*}$) into $z=g_{\phi }\left(f_{\theta^{*}}\left(\tilde{x}\right)\right)$.

### 2.2. CDAE-Based Denoising Model

A convolutional denoising autoencoder (CDAE) is an optimized version of a denoising autoencoder. In traditional denoising autoencoders, fully connected layers at each stage result in a substantial increase in computational requirements. Thus, in the convolutional denoising autoencoder, convolutional neural networks replace the fully connected networks. This replacement significantly reduces the number of parameters in the model and allows for the capture of local features in the signal.

**Figure 2:**
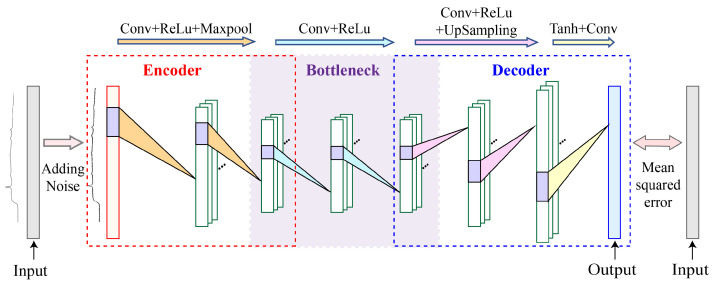
Architectural diagram of the CDAE denoising model.

Figure 2 illustrates the structure of our denoising model, which is based on a CDAE. In our CDAE-based denoising model, the encoder features two convolutional layers and two pooling layers. These convolutional and pooling layers work together to extract features and eliminate noise. Conversely, the decoder, with its convolutional and upsampling layers, expands the feature dimensions back to the size of the input data, reconstructing the denoised output. In this study, the typical convolutional denoising autoencoder is enhanced by the addition of two extra convolutional layers in its bottleneck. Typically, the bottleneck layer of an autoencoder comprises only the layer with the lowest dimensionality, which serves to maximally compress data and capture its core characteristics. Upon monitoring of the spectral signal-to-noise ratio (SNR) and mean square error (MSE), it was observed that a dual-layer encoding–decoding structure was inadequate to achieve the anticipated learning efficacy, indicating the necessity for further optimization of the model to enhance performance. Moreover, an analysis of the spectral data output by the model revealed that increasing the number of encoding–decoding layers could lead to excessive data compression, resulting in the loss of crucial spectral information. Therefore, to adequately extract and learn the features of spectral data, additional convolutional layers were introduced at the bottleneck stage of this model. This novel design is key to preserving essential information and enhancing the learning of spectral features. Additionally, our model incorporates an activation function after each convolutional layer to introduce nonlinearity. MSE is employed as the loss function in the model. The MSE effectively quantifies the difference between the model’s predictions and the actual data, guiding the optimization process towards more accurate denoising outcomes. The MSE is defined as follows:

(4) $$L\left(x,z\right)=\frac{1}{N}\sum_{i=1}^{N}{\left(x^{\left(i\right)}-z^{\left(i\right)}\right)}^{2}$$

where x represents the original spectrum, z denotes the predicted spectrum, and *N* refers to the length of the spectrum.

Finally, by extracting relevant data features and reconstructing the entire Raman spectrum, the CDAE model successfully retrieves Raman signals free from noise.

### 2.3. CAE-Based Baseline Correction Model

**Figure 3:**
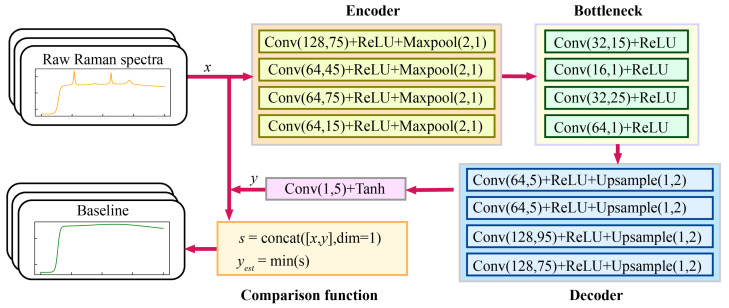
Architectural diagram of the CAE+ baseline correction model.

Unlike a convolutional denoising autoencoder (CDAE), convolutional autoencoders take original data as input rather than corrupted data with noise. CAEs can be used for unsupervised feature learning, data compression, and dimensionality reduction tasks. To capture the baseline features of the spectrum, a model named CAE+ was developed based on a convolutional autoencoder. This model is showcased in Figure 3. The CAE+ model can be divided into four parts, namely the encoder, decoder, bottleneck, and comparison function. The encoder contains four convolutional layers and four pooling layers, and this configuration is tailored to efficiently extract features from the spectrum. The decoder in the CAE+ model comprises four convolutional layers and four upsampling layers. This structure aids in reconstructing the baseline of the input spectrum efficiently. In the CAE+ model, the bottleneck includes four convolutional layers. This setup plays a crucial role in accurately capturing the baseline features of the spectrum. The comparison function module leverages the fact that baseline values in a spectrum are typically lower than signal values, aiding in effective baseline identification. Therefore, the comparison function is defined as follows:

(5) $$y_{\mathrm{est}}^{\left(i\right)}=\min{\left(x^{\left(i\right)},y^{\left(i\right)}\right)}^{a}\;\mathrm{for}\;{\left(i=1,2,\ldots ,N\right)}^{a}$$

where x represents the input Raman spectrum; y is the output of the decoder; *i* represents the *i*th data point in the signal; *N* refers to the length of the spectrum; and $y_{\mathrm{est}}$ represents the result of the model, which is the estimated baseline.

### 2.4. Evaluation Criteria

To assess our denoising model’s performance, we conducted tests on both simulated and measured spectra. This allowed us to compare its effectiveness with that of other prevalent Raman spectrum denoising algorithms.

#### 2.4.1. Evaluation on Simulated Spectra

In evaluating our denoising model’s performance on simulated spectra, two metrics were applied, namely the signal-to-noise ratio (SNR) and the root mean square error (RMSE). These metrics provided a comprehensive assessment of the model’s effectiveness in reducing noise while preserving the integrity of the spectral data. These metrics were used to compare the performance of our denoising model with that of other algorithms. For the baseline correction model, its effectiveness on simulated spectra was gauged using the RMSE metric.

(6) $$SNR=10\mathrm{lg}\left(\frac{\sum_{i=1}^{N}{\left(x_{\mathrm{ref}}^{\left(i\right)}\right)}^{2}}{\sum_{i=1}^{N}{\left(x^{\left(i\right)}-{\bar{x}}_{\mathrm{ref}}\right)}^{2}}\right)$$

(7) $$RMSE\left(x,x_{\mathrm{ref}}\right)=\sqrt{\frac{1}{N}\sum_{i=1}^{N}{\left(x^{\left(i\right)}-x_{\mathrm{ref}}^{\left(i\right)}\right)}^{2}}$$

where *x* represents the reconstructed spectrum, and $x_{\mathrm{ref}}$ represents the reference spectrum.

Besides the assessment of the spectrum, the changes in the Raman peaks after preprocessing were also evaluated. To precisely evaluate preprocessing effects, we calculated SNR and RMSE around the spectral peaks. The calculations were performed within a 2n+1 sample window centered on each peak $x_{k}$, where *n* is the full width at the half maximum of the Raman peak. This specific approach allows for a focused assessment of changes in the peak regions due to preprocessing.

#### 2.4.2. Evaluation on Measured Spectra

To comprehensively assess the noise reduction efficiency and preservation of peak characteristics across diverse preprocessing algorithms, the spectral signal-to-noise ratio (SSNR) was employed as a criterion, which is defined as follows:

(8) $$SSNR=\frac{{\bar{S}}_{p}}{\sigma_{n}}$$

where ${\bar{S}}_{p}$ represents the mean value of all Raman peak heights across the entire spectrum, and $\sigma_{n}$ is the standard deviation of the signal-free region.

## 3. Simulated and Experimental Spectra

### 3.1. Generation of Simulated Spectra

Raman spectra are composed of three components, namely Raman peaks $y_{r}$, a fluorescence background b, and random noise n. Therefore, the Raman spectrum can be expressed as $y=y_{r}+b+n$. The Raman peaks are generated by the superposition of multiple Lorentzian peaks [22,23].

(9) $$y_{r}=\sum_{i=1}^{m}\frac{2A_{i}}{\pi }\frac{w_{i}}{4{\left(x-x_{i}\right)}^{2}+w_{i}^{2}}$$

where *m* represents the number of Raman peaks, $x_{i}$ is the position of the *i*th peak along the horizontal axis, $w_{i}$ is the full width at the half maximum of the *i*th peak, and $A_{i}$ is the area under the curve from baseline.

**Figure 4:**
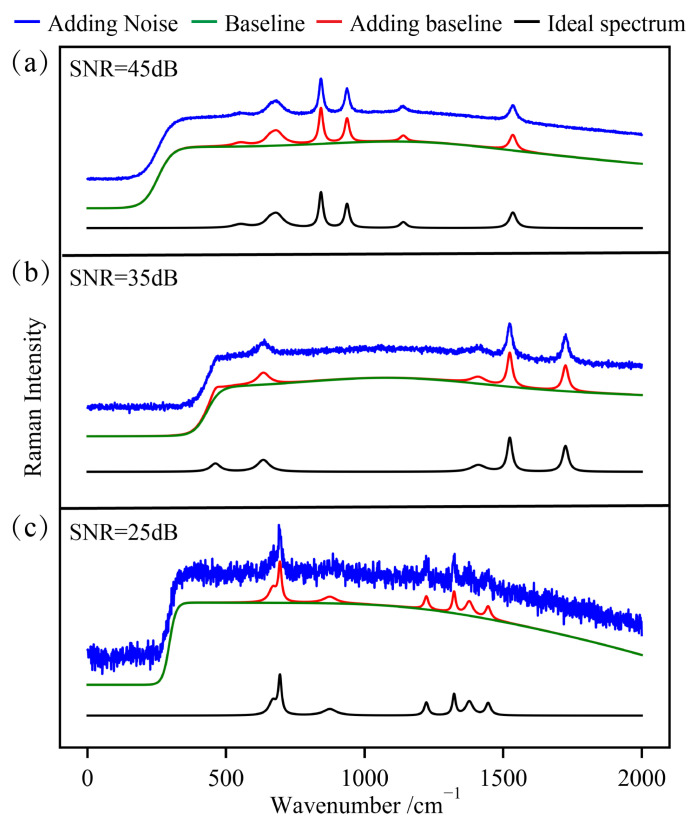
Simulated spectra with various noise levels and different baselines. (**a**) Simulated Raman spectrum with seven peaks at 552, 657, 683, 843, 937, 1140, and 1535 cm^−1^ and an SNR of 45 dB. (**b**) Simulated Raman spectrum with six peaks at 462, 635, 1398, 1412, 1524, and 1725 cm^−1^ and an SNR of 35 dB. (**c**) Simulated Raman spectrum with seven peaks at 669, 695, 875, 1223, 1323, 1446, and 1378 cm^−1^ and an SNR of 25 dB.

While this approach assumes symmetry in the peak shapes, the efficacy of our method in dealing with asymmetric peaks is substantiated through experimental spectra. To ensure the generality and effectiveness of our simulation analysis, we simulated the fluorescence background (b) using Gaussian, sigmoid, and polynomial baselines. These simulations were based on spectra collected by a Raman spectrometer, enhancing the reliability and applicability of the research results. The simulation spectral dataset constructed in this chapter comprises 3000 spectra, each consisting of 2000 data points, and includes 3 to 7 Raman peaks. The full width at the half maximum of each peak was set between 10 and 70 units, and the area of the Raman peaks varied from 1000 to 30,000. The simulated spectra are shown in Figure 4. The figure displays simulated ideal spectra, spectra with different baselines, and spectra with various levels of noise. We utilized these simulated spectra for model training.

### 3.2. Experimental Spectra

Experimental Raman spectra were collected with a Raman spectrometer system. The test setup comprises several critical components, each with a specific role. The spectrum stabilized laser, with a wavelength of 785 nm (IPS, Somerset, NJ, USA), acts as the excitation source, providing monochromatic light to induce Raman scattering. The high-throughput Raman spectrometer, coupled with a high-performance Raman-filtered fiber optic probe (EmVision LLC, Loxahatchee, FL, USA), detects the scattered light. It captures the Raman spectrum with high sensitivity and specificity and converts the optical signals into electrical signals for further analysis. A computer is also part of the setup; it is used for controlling the parameters of the spectrometer. The computer processes and analyzes the electrical signals received from the spectrometer, converting them into a digital form that can be used for the display and interpretation of the material’s molecular characteristics.

Beef fat tissue samples were purchased from a supermarket, and their spectra were collected for analysis. We set the laser power to 600 mW and the sampling time to 0.03 s. Single-scan, three-scan accumulation, and ten-scan accumulation modes were used to capture spectra at different noise levels in order to test the denoising capabilities of various algorithms. Then, the spectrum under ten-scan accumulation acquisition mode was selected to evaluate the effectiveness of different baseline correction algorithms.

## 4. Results and Discussion

### 4.1. Model Training and Evaluation

The learning rate is crucial for model training, serving as a key parameter that determines the speed and quality of the learning process. The learning rate of the CDAE model is $5\times 10^{-5}$, and the decay is 0.98. The learning rate of the CAE+ model is $1\times 10^{-4}$, and the decay is 0.98. In our training process, an early stopping mechanism was implemented. This function halts training if there is no decrease in validation loss for ten consecutive epochs. Such a strategy is crucial to prevent overfitting and save on training time.

**Figure 5:**
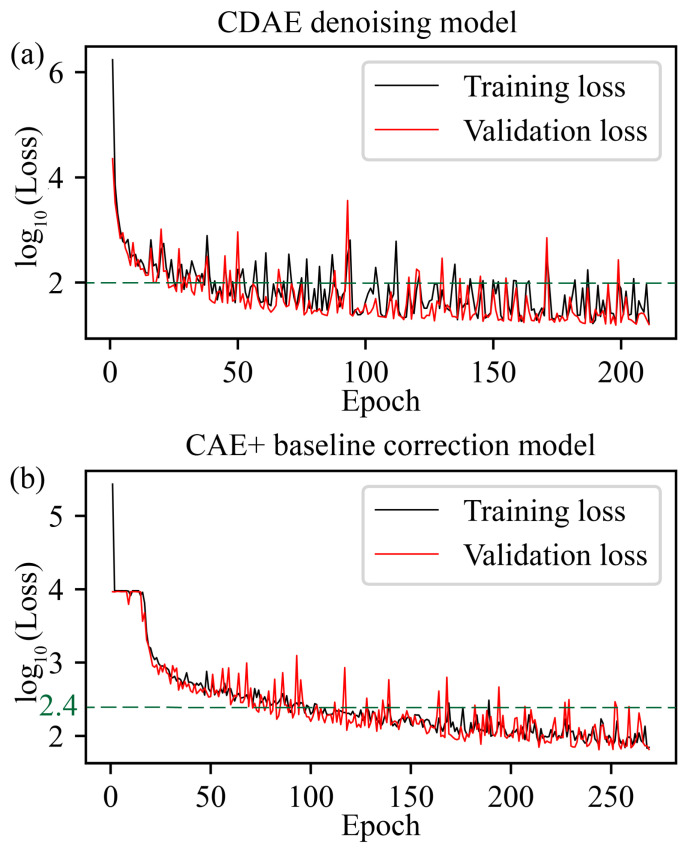
Training and validation loss (log10 scale) across epochs for (**a**) CDAE and (**b**) CAE+ models.

To more clearly present the training and validation loss values, we transformed them by taking their logarithms (base 10). The logarithmic transformations of the loss values for each epoch are displayed in Figure 5. Ideally, both training and validation loss decrease as training progresses, indicating that the model is learning and generalizing effectively. After 150 epochs of training, the logarithms of both the training and validation losses for the CDAE model generally stayed below 2. At the same time, in the CAE+ model, after 150 iterations, the logarithms of both the training and validation losses were almost maintained within 2.4.

These observations suggest that the models achieved a balance between generalization and fitting of the training data. The results validate the ability of the two models to identify data patterns effectively without overfitting, demonstrating their potential application in data analysis.

### 4.2. Evaluation of CDAE Denoising Model

#### 4.2.1. Simulation Spectra

To evaluate the performance of the CDAE denoising model, we compared it with traditional methods such as the Savitzky–Golay (SG) filter and the wavelet threshold denoising algorithm, demonstrating the effectiveness of the proposed method in various scenarios. In our comparisons, we optimized the SG filter with a window size of 35 data points. For wavelet threshold denoising, the optimized wavelet was set as Symlet 7 (Sym7). These specific settings were chosen to represent the capabilities of each method in our comparative analysis with the CDAE model.

For a thorough assessment of denoising performance on the spectrum with varying noise levels, we generated 60 simulated spectra, with 20 spectra each at SNR levels of 25 dB, 35 dB, and 45 dB, to represent varying noise conditions. We focused on assessing the performance of three different denoising algorithms, examining both their denoising capabilities and their ability to preserve Raman peak integrity within the spectra. We applied the three algorithms to denoise simulated spectra with varying noise levels. These tests were designed to evaluate the effectiveness of the denoising algorithms under various noise-level conditions. The average values of SNR and RMSE for these 20 spectra were calculated to provide a comprehensive overview. The results are detailed in Table 1.

Across the entire spectrum, the results reveal significant performance improvements by all three denoising algorithms over the original data. Notably, the CDAE model shows the most significant improvement in SNR, elevating the noise level of the spectra from 25 dB, 35 dB, and 45 dB to 36.93 dB, 47.99 dB, and 56.04 dB, respectively. This improvement proves that the CDAE model possesses superior signal preservation capability during denoising. Moreover, compared to the SG filter and wavelet thresholding denoising algorithms, the CDAE model stands out in reducing RMSE, indicating its ability to reconstruct spectral signals more accurately across various noise levels.

Observing the performance indicators in the Raman peak region from Table 1, it is evident that after introducing noise, the SNR and RMSE in the Raman peak areas of the spectrum perform better than those in other spectral regions. Both SNR and RMSE metrics significantly improved after applying the three denoising algorithms. However, compared to the performance across the entire spectrum, the improvements in the Raman peak regions were less pronounced. The CDAE model demonstrated superior denoising effects compared to the SG filter and Sym7 wavelet thresholding denoising, showcasing its effectiveness in noise reduction.

These findings suggest that the denoising efficacy of the algorithms within the Raman peak region is not as good as in the rest of the spectrum. When comparing the denoising effectiveness of the three algorithms in the Raman peak region, the CDAE model demonstrated superior performance, with the SNR improving to 36.92, 46.44, and 52.60 dB, respectively. Compared to the wavelet transformation and SG filtering methods, the CDAE model consistently showed lower RMSE and higher SNR across various noise levels in the spectra. This showcases its effectiveness in reducing noise.

#### 4.2.2. Experimental Spectra

**Figure 6:**
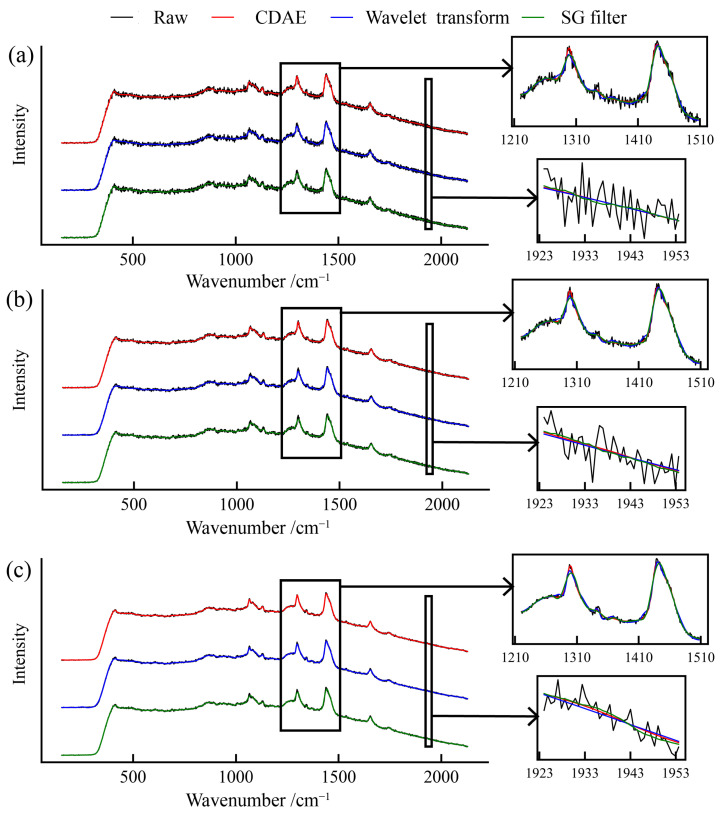
Denoising results of three algorithms under different modes: (**a**) single scan, (**b**) three-scan, and (**c**) ten-scan accumulation modes.

We used a spectrometer to collect Raman spectra under various scanning modes; these modes represent different levels of noise in the spectrum, allowing for a comprehensive analysis of the denoising algorithms under varying conditions. To clearly demonstrate the effects of peak preservation and denoising by the three algorithms, we zoomed-in the regions from 1210 to 1510 cm^−1^ and from 1923 to 1953 cm^−1^ in the spectrum, as shown in Figure 6.

Observing the range from 1923 to 1953 cm^−1^, the SG filter exhibited the largest signal fluctuations among the three algorithms after the denoising process. Comparatively, the CDAE model and the wavelet denoising algorithm showed smoother outcomes, suggesting more effective noise reduction in this frequency range.

In the region from 1210 to 1510 cm^−1^ of the spectrum, where two prominent Raman peaks are observed, both peaks are characterized by asymmetric shapes. The preservation of these peaks by three different denoising algorithms is compared in Figure 6. All three algorithms show similar accuracy in replicating the second peak. However, for the first peak, the wavelet denoising algorithm and the SG filter significantly reduce its intensity compared to the original spectrum. This is evident from the enlarged inset in Figure 6. In contrast, the CDAE model is more effective in maintaining the intensity of the Raman peak.

The 1040 to 1400 cm^−1^ region contains the Raman peaks of the sample signal. By testing four specific peaks within this area, the results demonstrate the effectiveness of three denoising algorithms in preserving the integrity of Raman peaks, as shown in Table 2. The peak preservation rate is defined as the ratio of Raman peak intensity in the denoised signal to that in the original signal, which is used to assess the integrity of Raman peaks.

The results reveal that the CDAE model’s peak preservation rates in the spectral range are between 0.851 and 0.96. The peak preservation rates of the CDAE consistently exceed the performance of both the SG filter and wavelet transformation in all spectral bands, highlighting its superior capability in maintaining peak integrity. The CDAE model consistently outperforms the other two algorithms in peak preservation across all regions and shows an approximately 10% higher peak preservation rate in the most noisy region. It is concluded that the CDAE model possesses a greater effectiveness in preserving peaks.

Overall, the CDAE model outperforms the SG filter and wavelet denoising in both peak intensity preservation and overall denoising. This demonstrates its effectiveness and reliability as a denoising tool, showcasing outstanding performance in spectral data processing under various noise conditions.

### 4.3. Evaluation of CAE+ Baseline Correction Model

#### 4.3.1. Simulation Spectra

We generated 50 spectra with different baselines to test the performance of baseline correction algorithms. We calculated the RMSE for each algorithm across the entire spectrum and the Raman peak region. We conducted statistical analysis on the RMSE of the 50 spectra, including the calculation of the mean (mean) and standard error (SE), with results presented in Table 3. These metrics were used to quantify the performance of each algorithm in baseline correction.

In the overall spectral analysis, the CAE+ model showed significant superiority over the other three algorithms. It achieved lower averages and standard error in RMSE, indicating enhanced baseline correction precision and consistency. Notably, compared to the CAE model, the CAE+ model reduced the mean from 12.6 to 7.6 and decreased the standard error from 58.5 to 22.3. This marked improvement highlights the role of the comparison function in the CAE+ model. It significantly improves the fitting accuracy and reliability of the baseline across the spectral range.

In the Raman peak region, the analysis reveals that both airPLS and iterative polynomial fitting algorithms exhibit high means and standard deviations, as shown in the second row of Table 3. This suggests that the two algorithms have lower accuracy in baseline fitting within this region. Notably, compared to their performance across the full spectrum, these two algorithms show a significant increase in the mean and standard error in the peak area. This indicates a reduction in their baseline correction capability within the Raman peak region. In contrast, the CAE and CAE+ models both effectively correct baselines in the Raman peak region. Their mean and standard error in this specific area are consistent with their performance across the entire spectrum. This contrasts with the higher errors seen with airPLS and iterative polynomial fitting in this region. Notably, the performance of the CAE+ model surpasses that of the CAE model overall, underscoring the added effectiveness of the comparison function in the CAE+ model for enhanced baseline correction throughout the entire spectrum.

#### 4.3.2. Experimental Spectra

**Figure 7:**
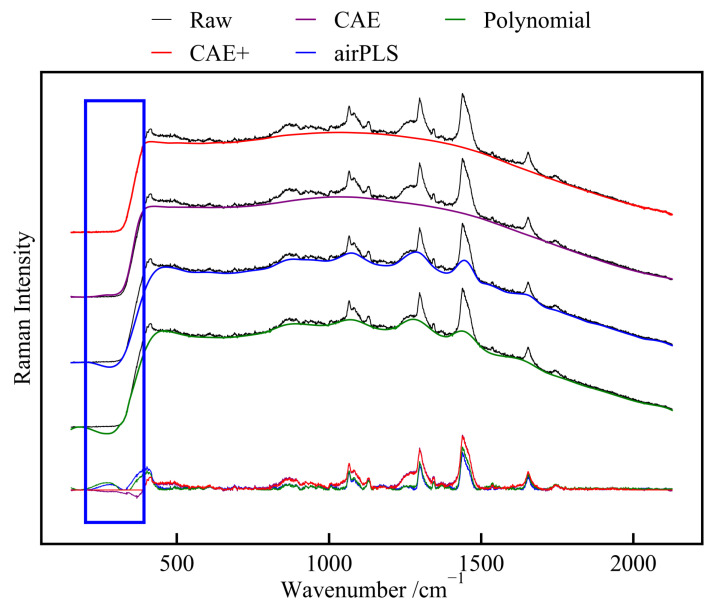
Baseline correction results of different algorithms on spectra collected in ten-scan acquisition mode.

To evaluate the baseline correction performance of different algorithms, we utilized spectra that were collected using a ten-scan acquisition mode. The results of this testing, showcasing the performance of each algorithm, are illustrated in Figure 7. The figure presents the baselines as fitted by the four different algorithms, providing a visual comparison of the spectral signals after baseline correction.

In the range from 200 to 392 cm^−1^ (highlighted by a blue box), which represents the background baseline, noticeable differences are observed in the algorithms’ performance. The CAE model displays a downward peak in this region. At the same time, the baselines fitted by both the iterative polynomial fitting and airPLS algorithms deviate significantly from the actual background baseline, even introducing an extra peak, indicating variances in their baseline fitting accuracy. In comparison, the CAE+ model accurately estimates the baseline in this region. The corrected signal is flat, with no inverted peaks or additionally introduced feature peaks.

Additionally, the fitting of baselines by the airPLS and iterative polynomial algorithms leads to a reduction in the intensity of Raman peaks. This suggests that these algorithms may inadvertently diminish peak strength during their baseline correction processes. In contrast, the CAE and CAE+ models effectively retain the complete Raman peaks.

To further quantify the impact of different baseline correction algorithms on Raman peak intensity, we utilized signals with manually subtracted baselines as a benchmark for assessment and comparison. We calculated the ratio of the Raman peak intensity corrected by various algorithms to the intensity of the manually baseline-subtracted reference signal and designated the ratios as peak preservation rates. The peak preservation rates were calculated for the CAE+ model, airPLS, and iterative polynomial fitting, with the results displayed in Table 4, containing the peak preservation rates for four prominent Raman peaks within the 1040 to 1400 cm^−1^ region.

In all four Raman peaks, the iterative polynomial fitting and airPLS algorithms show lower peak preservation rates—from 0.66 to 0.782 and from 0.72 to 0.919, respectively. Notably, they perform better in the 1350 to 1400 cm^−1^ band, indicating that these algorithms are more effective at preserving peak intensity in narrower peaks compared to broader ones. At the same time, the CAE+ model’s peak preservation rate is close to 1 across all bands, indicating that the post-correction peak intensity nearly matches the original. This showcases the CAE+ model’s high accuracy in preserving peak intensities during baseline correction. Overall, the CAE+ model effectively addresses the challenges faced by classic algorithms in preserving Raman peak intensities during baseline correction, making it more accurate and stable for spectra collected by experimental instruments.

### 4.4. Preprocessing Results: CDAE Model Combined with CAE+ Model

**Figure 8:**
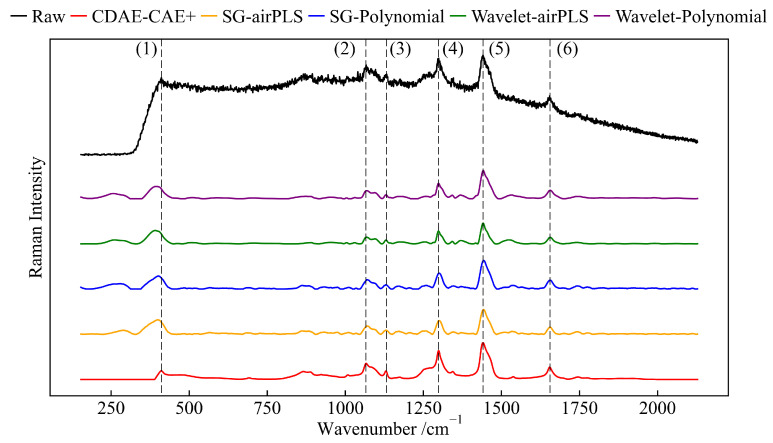
Results of different preprocessing algorithms on measured spectra.The peak positions are (1) 411 cm^−1^, (2) 1071 cm^−1^, (3) 1132 cm^−1^, (4) 1296 cm^−1^, (5) 1440 cm^−1^ and (6) 1656 cm^−1^.

To validate the effectiveness of the CDAE-CAE+ model, we tested it on spectra from beef fat tissue samples, comparing it with traditional preprocessing methods. The CDAE-CAE+ model was implemented by first employing the CDAE to reduce the noise in the data, followed by the application of CAE+ to perform baseline correction. The outcomes of this comparison are presented in Figure 8. From the figure, it is evident that classic preprocessing algorithms caused reductions in the intensity and transformations in the shapes of Raman peaks. In particular, methods based on SG filtering resulted in shifts in the positions of the second and fifth Raman peaks, as indicated in the figure. In contrast, the CDAE-CAE+ model significantly reduces noise, and there are minimal fluctuations in the spectral bands without signals. It effectively preserves the fundamental characteristics of the original signal peaks, such as their shape, position, and height, highlighting its efficiency in spectral analysis.

We calculated the SSNR for each signal processed by the five preprocessing methods. For this, we used noise signals derived from the 1980 to 2127 cm^−1^ range (the last two hundred data points) and the second to sixth Raman peaks marked in the figure. The result is presented in Table 5. The SSNR of our proposed model significantly surpasses those of the other four algorithms. The model demonstrates superior noise reduction capabilities. Additionally, it effectively retains the integrity of the signal during data processing, ensuring high-quality spectral analysis.

## 5. Conclusions

This paper introduces a set of preprocessing methods based on a convolutional autoencoder for denoising and baseline correction. Validated through both simulated and measured spectra, our study effectively showcases the performance of the CDAE-CAE+ model in preprocessing Raman spectra. Experimental results indicate its superior performance over traditional methods during denoising and baseline correction. The CDAE model can improve denoising capability and peak preservation ability, and the CAE+ model can retain the original Raman peak intensities and shapes. Compared to other preprocessing methods, the CDAE-CAE+ model not only enhances the spectral signal-to-noise ratio but also preserves the original shape, intensity, and position of Raman peaks. The proposed method was validated by simulated and experimental results, demonstrating its potential application for practical Raman spectral analysis.

## Figures and Tables

**Table 1:** Comparison of denoising results across various algorithms on simulated spectra.

Region	Algorithm	RMSE			SNR		
Entire	Raw	85.66	26.22	9.22	25	35	45
	CDAE	21.80	5.86	2.55	36.93	47.99	56.04
	SG	27.84	10.20	3.88	34.75	43.38	52.92
	Sym7	22.56	8.30	3.17	36.22	44.94	54.30
Peaks	Raw	85.39	26.01	9.30	26.56	36.59	46.30
	CDAE	25.93	8.44	4.67	36.92	46.44	52.60
	SG	31.18	13.74	5.65	35.48	43.20	51.78
	Sym7	30.53	11.14	5.18	35.78	44.06	51.51

**Table 2:** Peak preservation rates of various denoising algorithms under different acquisition modes.

Mode	Algorithm	Band (cm^−1^)			
		1040–1110	1210–1340	1400–1510	1350–1400
Single	CDAE	0.890	0.918	0.906	0.855
	Sym7	0.747	0.796	0.878	0.629
	SG	0.782	0.786	0.875	0.709
Three	CDAE	0.851	0.914	0.916	0.955
	Sym7	0.706	0.815	0.899	0.760
	SG	0.741	0.786	0.888	0.867
Ten	CDAE	0.880	0.953	0.960	0.952
	Sym7	0.826	0.854	0.959	0.741
	SG	0.749	0.818	0.938	0.781

**Table 3:** Results comparison of various baseline correction algorithms.

	airPLS		Polynomial		CAE		CAE+	
	Mean	SE	Mean	SE	Mean	SE	Mean	SE
Entire	61.6	435.4	71.6	388.4	58.5	12.6	7.6	22.3
Peaks	124.9	1762	166.2	2739	76.4	12.4	11.3	83.8

**Table 4:** Peak preservation rate results for various baseline correction algorithms.

Algorithm	Band (cm^−1^)			
	1040–1110	1210–1340	1400–1510	1350–1400
CAE+	0.997	0.997	1.033	0.985
airPLS	0.660	0.644	0.743	0.782
Polynomial	0.720	0.724	0.852	0.919

**Table 5:** Spectral signal-to-noise ratio comparison for different preprocessing algorithms.

Noise Reduction	Baseline Correction	SSNR
CDAE	CAE	553.1
SG	airPLS	68.8
SG	Polynomial	61.8
Sym7	airPLS	85.3
Sym7	Polynomial	59.3

## Data Availability

The data is available upon request.
